# Spatiotemporal dynamics of hemorrhagic fever with renal syndrome in Jiangxi province, China

**DOI:** 10.1038/s41598-020-70761-0

**Published:** 2020-08-31

**Authors:** Shu Yang, Yuan Gao, Xiaobo Liu, Xiaoqing Liu, Yangqing Liu, Soeren Metelmann, Chenying Yuan, Yujuan Yue, Shengen Chen, Qiyong Liu

**Affiliations:** 1 The Collaboration Unit for Field Epidemiology of State Key Laboratory of Infectious Disease Prevention and Control, Nanchang Center for Disease Control and Prevention, Nanchang, 330038 China; 2 State Key Laboratory of Infectious Disease Prevention and Control, National Institute for Communicable Disease Control and Prevention, Chinese Center for Disease Control and Prevention, Beijing, 102206 China; 3 Institute of Infection and Global Health, University of Liverpool, Liverpool, L69 7BE UK

**Keywords:** Environmental sciences, Environmental social sciences, Diseases, Mathematics and computing

## Abstract

Historically, Jiangxi province has had the largest HFRS burden in China. However, thus far, the comprehensive understanding of the spatiotemporal distributions of HFRS is limited in Jiangxi. In this study, seasonal decomposition analysis, spatial autocorrelation analysis, and space–time scan statistic analyses were performed to detect the spatiotemporal dynamics distribution of HFRS cases from 2005 to 2018 in Jiangxi at the county scale. The epidemic of HFRS showed the characteristic of bi-peak seasonality, the primary peak in winter (November to January) and the second peak in early summer (May to June), and the amplitude and the magnitude of HFRS outbreaks have been increasing. The results of global and local spatial autocorrelation analysis showed that the HFRS epidemic exhibited the characteristic of highly spatially heterogeneous, and Anyi, Fengxin, Yifeng, Shanggao, Jing’an and Gao’an county were hot spots areas. A most likely cluster, and two secondary likely clusters were detected in 14-years duration. The higher risk areas of the HFRS outbreak were mainly located in Jiangxi northern hilly state, spreading to Wuyi mountain hilly state as time advanced. This study provided valuable information for local public health authorities to design and implement effective measures for the control and prevention of HFRS.

## Introduction

Hemorrhagic fever with renal syndrome (HFRS) is a rodent-borne infectious disease caused by hantaviruses in the *Bunyaviridae* family^1^. Transmission of hantavirus to humans occurs via inhalation of aerosolized viral particles present in the urine, feces, and saliva excreted into the environment by rodents infected with it^2^. In China, the major causative agents of HFRS are Hantaan virus (HTNV) and Seoul virus (SEOV), whose natural rodents hosts are respectively striped field mice (*A. agrarius*) and Norway rats (*R. norvegicus*)^3,4^. So far, China remains the most endemic country, and there were more than 11,000 HFRS cases reported annually from 2016 to 2018^5^.

Jiangxi province, which is located in the southern bank of the middle and lower reaches of the Yangzi River, is one of the most serious HFRS endemic areas of China. Since the first case of HFRS was reported in Pengze county in 1961, the HFRS epidemic has rapidly spread to 6 counties in the 1960s, 39 counties in the 1970s, 65 counties in the 1980s, and 88 counties in the 1990s. The number of HFRS cases has also risen sharply, and reached a peak in 1985, with an incidence of 21/100,000 persons. The epidemic of HFRS has expanded throughout the central and northern Jiangxi and reached Ningdu county, Ganzhou city, in the south^6^. Jiangxi currently remains one of the provinces with the highest HFRS incidence during recent years according to the national HFRS surveillance data^7^.

The susceptible population of HFRS is heterogeneous in space, socioeconomic status, and the geographical difference between different regions^8^. Furthermore, infectious disease outbreaks can occur in very short time periods and infect a large number of individuals^9,10^. As a result, the adequate distinction between incidence classes can play a significant role in the accurate mapping and risk assessment of regional disease spread during the time period of interest^11–13^. Spatiotemporal analysis has the power of quantitative statistics and mapping visualization, and has been also widely conformed to characterize spatial epidemiology of diseases^14–19^. However, the dynamics spatiotemporal distributions of HFRS in Jiangxi have not yet been explored systematically. So this study aims to explore the dynamics of spatiotemporal distributions based on the case surveillance data from 2005 to 2018 at the county scale in Jiangxi, and providing valuable scientific support for HFRS monitoring and control.

## Materials and methods

### Study areas

**Figure 1:**
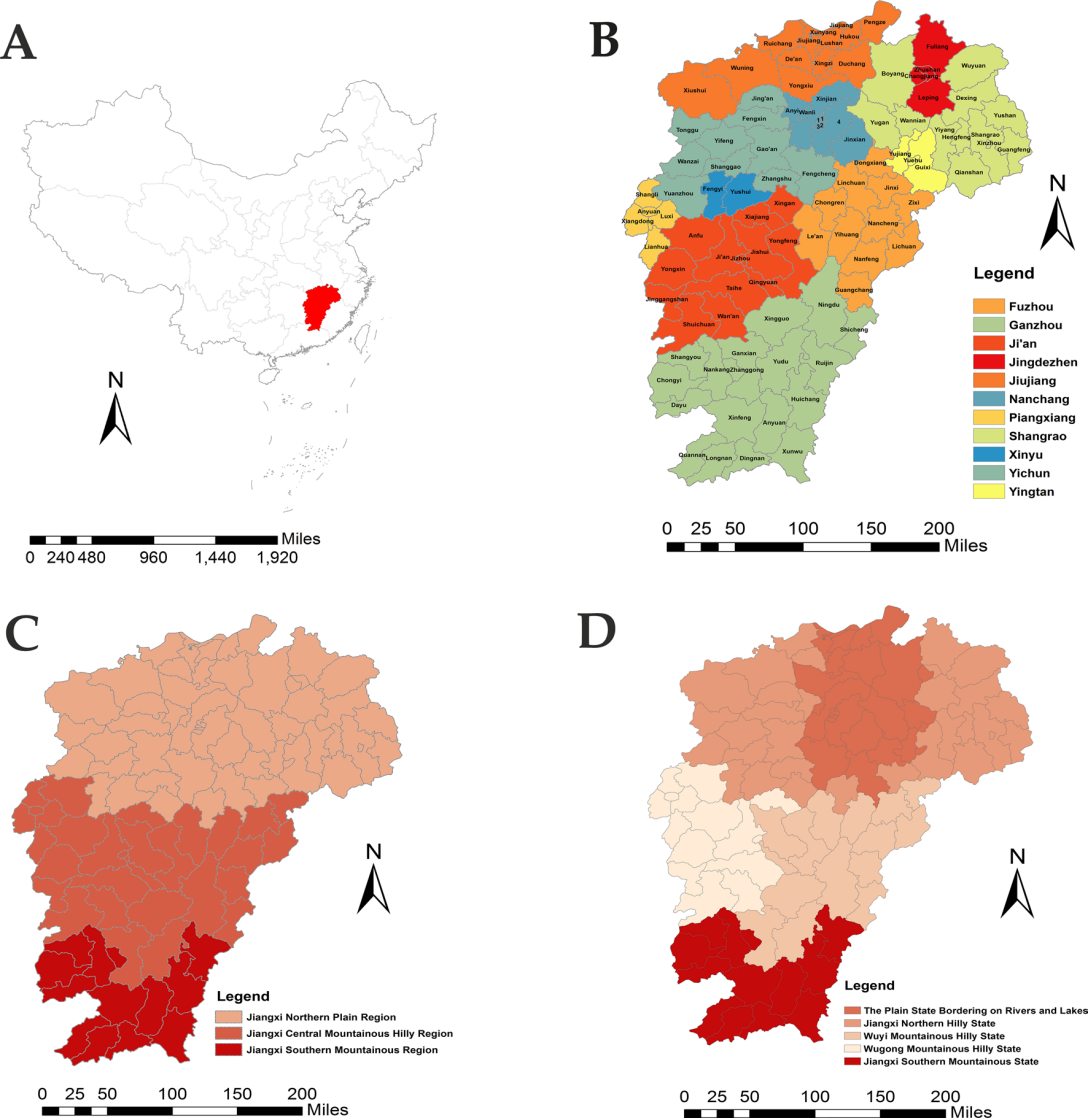
The location of the study area. (**A**) Location of Jiangxi province, in China. (**B**) Administrative division of the study area(1. Donghu; 2. Xihu; 3. Qingyunpu; 4. Nanchang county). (**C**) The geographic distribution of three zoogeographic regions. (**D**) The geographic distribution of five zoogeographic states. These maps were generated by ArcGIS software (Version 10.4 ESRI, Redlands, CA, USA, https://www.esri.com/software/arcgis/arcgis-for-desktop).

Jiangxi (24°29′14″–30°04′44″ N, 113°34′36″–118°28′58″ E) lies in southeastern China, with an approximate area of 16.5 thousand km^2^, and population of 44.56 million in 2010, including 11 cities and 100 counties (Fig. 1). Jiangxi belongs to a humid subtropical climate, with annual rainfall, annual average temperature, and annual average sunshine ranging from 1,341 to 1,943 mm, 16.2 to 19.7 °C, 1,473 to 2,077 h, respectively^20^.

Jiangxi is surrounded by mountains on three sides and facing the Yangtze River on the other. The southern half of the province is hills with ranges and valleys interspersed; while the middle and northern half is flatter and lower in altitude. Stretching from south to north, the whole land is generally sloping towards Poyang lake, which has formed a huge basin opening to the north^20^. In historical literatures, Jiangxi was often divided into three zoogeographic regions and five zoogeographic states: Jiangxi northern plain region (including the plain state bordering on rivers and lakes and Jiangxi northern hilly state), Jiangxi central mountainous hilly region (including Wuyi mountainous hilly state and Wugong mountainous hilly state), and Jiangxi southern mountainous region (including Jiangxi southern mountainous state) (Fig. 1).

### Data source

Reported daily HFRS data for the period of 2005 to 2018 were extracted from CISDCP in the Chinese Center for Disease Control and Prevention (China CDC). The gathered information about individual HFRS cases included the age, occupation onset and confirmation date, case category, and residential address. The diagnosis of HFRS cases referred to the ‘Diagnostic criteria for epidemic hemorrhagic fever’ (WS278–2008) of China (https://www.nhc.gov.cn/wjw/s9491/200802/39043.shtml). HFRS cases were aggregated and geocoded to the corresponding county in ArcGIS (Version 10.4, ESRI Inc., Redlands, CA, USA). The base map was acquired from the geospatial data cloud (https://www.gscloud.cn/). The population size in every county was issued by the National Bureau of Statistics of the People’s Republic of China (https://www.stats.gov.cn/tjsj/ndsj/). Clinically diagnosed and laboratory-confirmed cases were included in our study. And 148 cases were excluded due to invalid addresses (incomplete, incorrect or outside study area) or suspected cases.

### Spatiotemporal cluster analysis

A seasonal-trend decomposition of time series analysis was performed to explore the various characteristics of periodicity and seasonality in R software (Version 3.1 AT&T BellLaboratories, Auckland, New Zealand). A global spatial autocorrelation analysis and a Local Indicators of Spatial Association (LISA) analysis were conducted in ArcGIS software (Version 10.4, ESRI Inc., Redlands, CA, USA) to visualize the global and local spatial clustering of HFRS cases in Jiangxi from 2005 to 2018. Global Moran’s *I* Index (ranged from − 1 to 1) was used to analyze global spatial autocorrelation. Moran’s Index = 0 implied a random spatial distribution. Moran’s *I* Index < 0 implied a dispersing spatial distribution, and Moran’s *I* Index > 0 implied a clustering spatial distribution. Local Moran’s *I* was calculated to explore significant hot spots (High–High), cold spots (Low–Low), and outliers (High–Low and Low–High)^21^. A Kulldorff’s spatiotemporal scan statistical analysis was used to identify the spatiotemporal clusters of HFRS cases in Jiangxi in SaTScan Software (Version 9.4, Martin Kulldorff, National Cancer Institute, Bethesda, MD, USA)^22^. The discrete Poisson probability model by a circular window with a radius was used for scanning. The maximum of the spatial and temporal size were all defined as 25%. A *P* < 0.05 was considered to be significant.

The maps were made in ArcGIS software (Version 10.4, ESRI Inc., Redlands, CA, USA).

### Ethics approval

Ethical approval for the research was granted by the Chinese Center for Disease Control and Prevention Ethics Committee (No. 2012CB955504). This study didn’t collect patients’ samples, and the data obtained from the China Information System for Disease Control and Prevention (CISDCP) were anonymized so that subjects could not be identified. Therefore, the ethics committee agreed that no informed consent was needed from patients. All methods in our study were used in accordance with the relevant guidelines and regulations.

## Results

### Descriptive statistics

A total of 7,203 HFRS cases with a case fatality rate of 1.34%, were reported in Jiangxi from 2005 to 2018. The annual case number ranged from 335 in 2009 to 683 in 2018, and presented a total uptrend during the 14-year period. Male cases were 4,950 (68.7%), and the male-to-female ratio (not significant) ranged from 2.9:1 in 2008 to 1.9:1 in 2018. A total of 74.4% of HFRS cases occurred in individuals aged from 16 to 60 years old, and different age group ratios of different years were significantly different (χ^2^ = 217.0, *p* = 0.000). The majority of HFRS cases were farmers (67.2%), followed by students (8.4%). Different occupational ratios of different years existed significant differences (χ^2^ = 180.7, *p* = 0.000) (Table 1).

**Table 1:** Characteristics of all 7,203 hemorrhagic fever with renal syndrome cases in the study areas, 2005–2018.

Year	Gender		Occupation					Age		
	Male	Female	Students	Farmers	Workers	Housework/unemployment	Others	< 16	16~	> 60
2005	308 (68.6)	141 (31.4)	53 (11.8)	289 (64.4)	21 (4.7)	25 (5.6)	61 (13.6)	40 (9.1)	374 (83.3)	35 (8.0)
2006	292 (71.0)	119 (29.0)	36 (8.8)	275 (66.9)	31 (7.5)	28 (6.8)	41 (10.0)	22 (5.4)	345 (83.9)	44 (10.8)
2007	271 (70.9)	111 (29.1)	33 (8.6)	285 (74.6)	24 (6.3)	9 (2.4)	31 (8.1)	20 (5.3)	320 (83.8)	42 (11.2)
2008	237 (67.5)	81 (29.1)	26 (7.4)	268 (76.4)	17 (4.8)	13 (3.7)	27 (7.7)	20 (5.7)	296 (84.3)	35 (10.0)
2009	244 (72.8)	91 (27.2)	28 (8.4)	235 (70.1)	16 (4.8)	20 (6.0)	36 (10.7)	18 (5.5)	277 (82.7)	40 (12.1)
2010	262 (68.6)	120 (31.4)	35 (9.2)	247 (64.7)	25 (6.5)	22 (5.8)	53 (13.9)	29 (7.6)	314 (82.2)	39 (10.3)
2011	356 (68.2)	166 (31.8)	38 (7.3)	361 (69.2)	21 (4.0)	32 (6.1)	70 (13.4)	36 (7.0)	410 (78.5)	76 (14.8)
2012	437 (73.0)	162 (27.0)	39 (6.5)	429 (71.6)	13 (2.2)	52 (8.7)	66 (11.0)	42 (7.0)	432 (72.1)	125 (20.9)
2013	454 (66.6)	228 (33.4)	65 (9.5)	461 (67.6)	21 (3.1)	67 (9.8)	68 (10.0)	65 (9.7)	479 (70.2)	138 (20.5)
2014	354 (69.7)	154 (30.3)	29 (5.7)	333 (65.6)	28 (5.5)	47 (9.3)	71 (14.0)	33 (6.5)	376 (74.0)	99 (19.6)
2015	447 (66.1)	229 (33.9)	61 (9.0)	439 (64.9)	22 (3.3)	43 (6.4)	111 (16.4)	63 (9.4)	471 (69.7)	142 (21.1)
2016	454 (67.9)	215 (32.1)	54 (8.1)	426 (63.7)	17 (2.5)	60 (9.0)	112 (16.7)	63 (9.4)	449 (67.1)	157 (23.5)
2017	389 (70.2)	165 (29.8)	52 (9.4)	334 (60.3)	21 (3.8)	60 (10.8)	87 (15.7)	57 (10.3)	376 (67.9)	121 (21.9)
2018	445 (65.2)	238 (34.8)	58 (8.5)	456 (66.8)	17 (2.5)	79 (11.6)	73 (10.7)	71 (10.4)	438 (64.1)	174 (25.5)
Total	4,950 (68.7)	2,253 (31.3)	607 (8.4)	4,838 (67.2)	294 (4.1)	557 (7.7)	907 (12.6)	579 (8.0)	5,357 (74.4)	1,267 (17.6)

**Figure 2:**
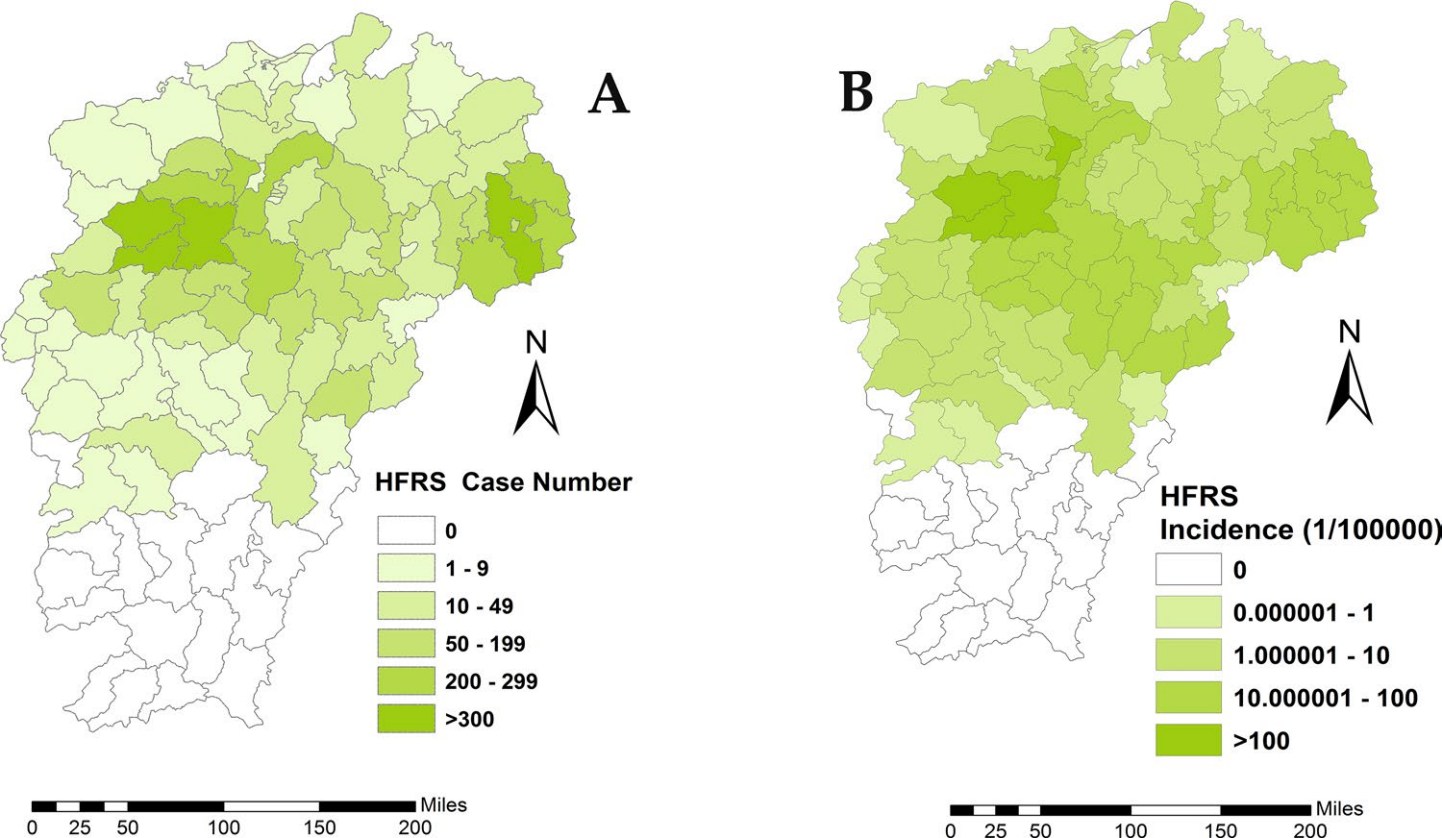
Spatial distribution of hemorrhagic fever with renal syndrome cases in the study area. (**A**) Spatial distribution of HFRS case. (**B**) Cumulative incidence of HFRS case. These maps were generated by ArcGIS software (Version 10.4 ESRI, Redlands, CA, USA, https://www.esri.com/software/arcgis/arcgis-for-desktop).

The cumulative number of HFRS cases in each county ranged from 0 to 1,060, among which Shanggao county had the highest annual number of 284 cases. The cumulative incidence rate of HFRS varied between counties (Fig. 2). The top ten counties with the highest HFRS incidence rates were Shanggao (324.46/100,000), Yifeng (290.46/100,000), An’yi (135.41/100,000), Gao’an (120.87/100,000), Fengxin (88.40/100,000), Qianshan (66.46/100,000), Hengfeng (63.47/100,000), Yushan (58.96/100,000) and Xingan (50.32/100,000), all of which located in Jiangxi northern hilly state. No cases were reported from Jiangxi southern mountainous state.

### Seasonal decomposition analyses

**Figure 3:**
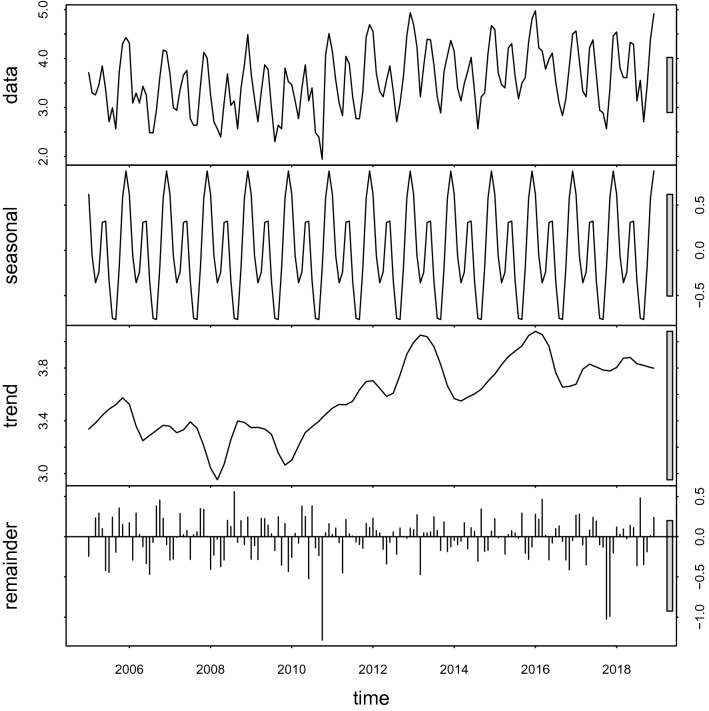
Decomposed hemorrhagic fever with renal syndrome cases in the study area from 2005 to 2018. These maps were generated by ArcGIS software (Version 10.4 ESRI, Redlands, CA, USA, https://www.esri.com/software/arcgis/arcgis-for-desktop).

The seasonal trend was explored using a season-trend decomposition in R software (Fig. 3). There was a rhythmic vibration in the raw data from 2005 to 2018. Seasonality and trend components were isolated from the raw data and also eliminated part of the random noise or reminder component. The epidemic of HFRS showed a bi-peak seasonality characteristic each year, with the primary peak occurring in winter (November to January) and the second peak happening in early summer (May to June). In addition, the epidemic of HFRS collectively showed an upward trend and the variation characteristic of periodicity with the amplitude and the magnitude of the periodical variation increasing.

### Spatial autocorrelation analysis

The global spatial autocorrelation analysis was performed based on county-level HFRS cases in Jiangxi (Table 2). The annual global Moran’s *I* indexes, which ranged from 0.14 to 0.38, all passed the significance level test (*p* < 0.05), indicating that the spatial distribution of the HFRS epidemic was not random from 2005 to 2018, with t-shaped spatial autocorrelation characteristics at county-level scale. Moran’s *I* indexes showed an uptrend from 2006 to 2010, and then stayed at a relatively stable level after 2011, suggesting that the spatial aggregation increased first and then stabilized.

**Table 2:** Global spatial autocorrelation analysis of reported hemorrhagic fever with renal syndrome in the Jiangxi province of China, 2005–2018.

Year	Moran’s *I*	*Z*-value	*P*-value
2005	0.25	2.77	< 0.01
2006	0.14	2.01	< 0.05
2007	0.22	2.65	< 0.01
2008	0.29	3.15	< 0.01
2009	0.25	2.80	< 0.01
2010	0.38	3.78	< 0.001
2011	0.33	3.30	< 0.001
2012	0.33	3.41	< 0.001
2013	0.31	3.20	< 0.01
2014	0.30	3.49	< 0.001
2015	0.32	3.54	< 0.001
2016	0.27	3.22	< 0.01
2017	0.38	3.86	< 0.001
2018	0.34	3.58	< 0.001
2005–2018	0.35	3.62	< 0.001

**Figure 4:**
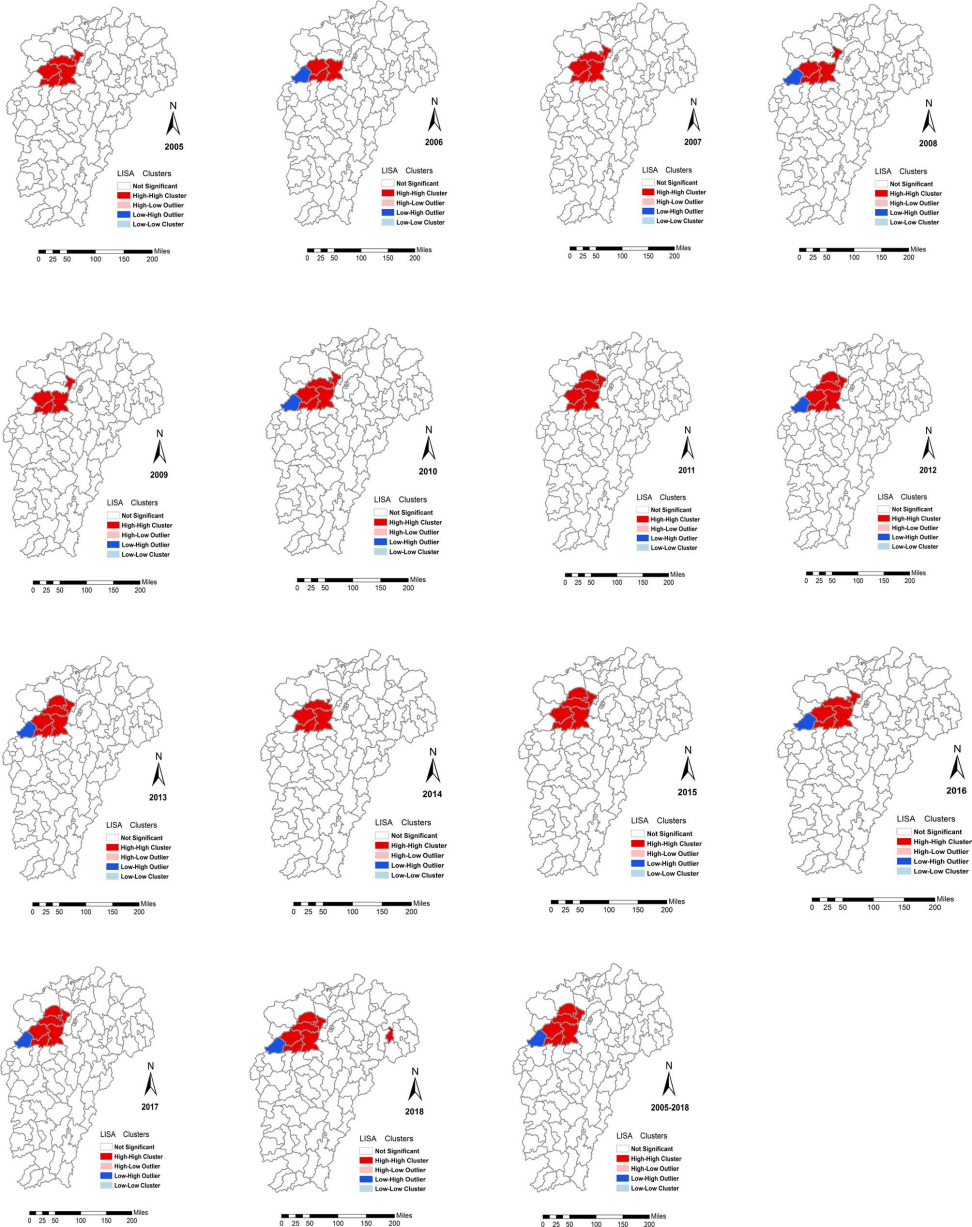
Local indicators of spatial association cluster maps for hemorrhagic fever with renal syndrome in the study area from 2005 to 2018. These maps were generated by ArcGIS software (Version 10.4 ESRI, Redlands, CA, USA, https://www.esri.com/software/arcgis/arcgis-for-desktop).

LISA was performed to detect local spatial clusters of the HFRS epidemic (Fig. 4). Three counties, Yifeng, Shanggao and Gao’an, were hot spots (High–High cluster area) in the whole study period and An’yi was a relatively stable hot spot. Moreover, Fengxin became a stable hot spot after 2010, and Jing’an developed into a relatively stable hot spot since 2011, and Hengfeng was identified as a hot spot in 2018, indicating that hot spots areas expanded gradually. Wanzai was an outlier (Low–High cluster area). All of them were in the eastern part of Jiangxi northern hilly state.

### Spatiotemporal clusters analysis

**Figure 5:**
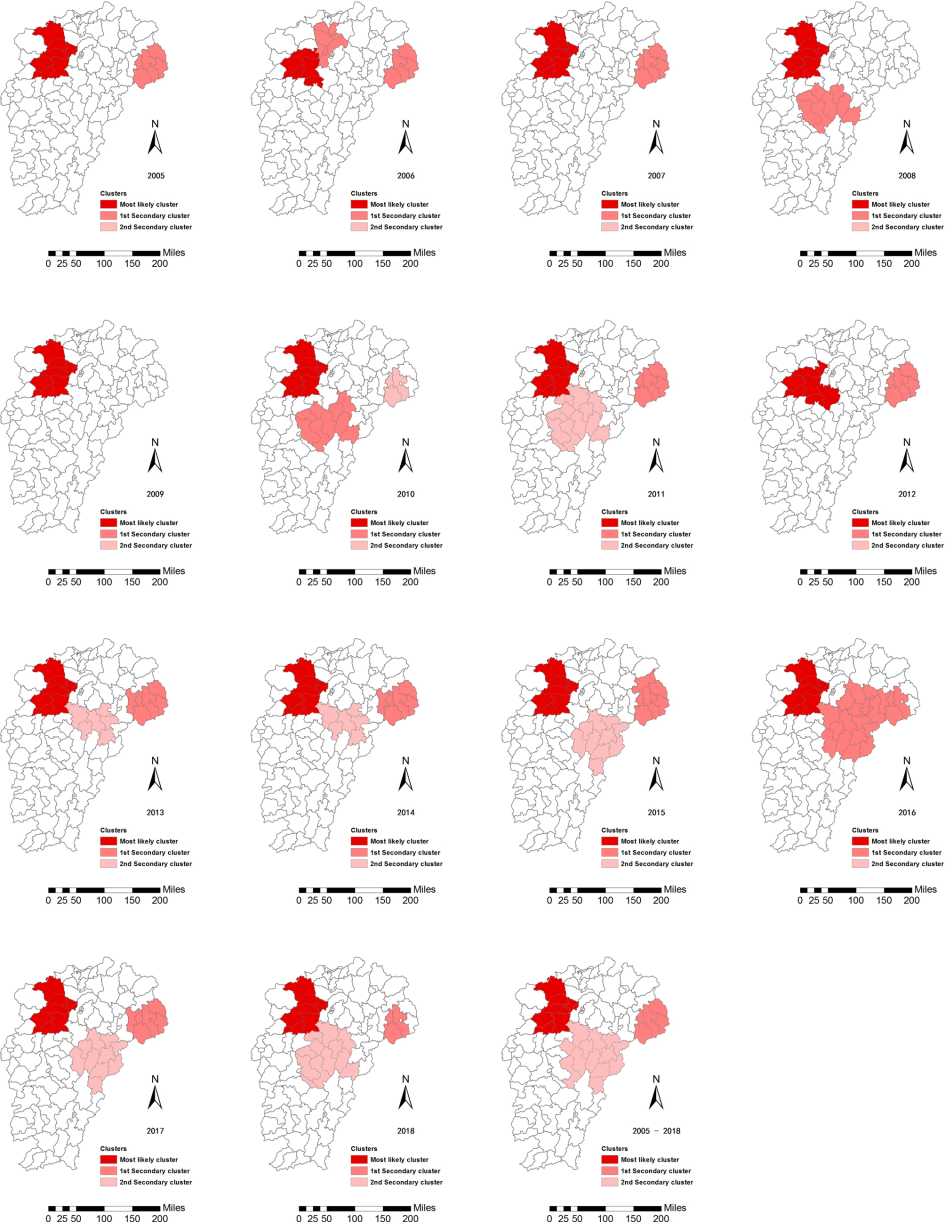
Yearly spatiotemporal clusters of hemorrhagic fever with renal syndrome cases in the study area from 2005 to 2018 using Kulldorff’s space–time scan statistic. These maps were generated by ArcGIS software (Version 10.4 ESRI, Redlands, CA, USA, https://www.esri.com/software/arcgis/arcgis-for-desktop).

The incidence of HFRS was aggregated through space and time using Kulldorff’s spatiotemporal scan statistics. The distribution of the HFRS epidemic existed significant heterogeneity in Jiangxi from 2005 to 2018. The most likely cluster was stable on the whole located in the western part of the Jiangxi northern hilly state, and showed the trend of spreading to the periphery in 2006 and 2012. Secondary clusters formed a core area in the eastern part of Jiangxi northern hilly state, spread to Wuyi mountain hilly state over time, firstly developed into two isolated parts in 2010, and merged into the largest one in 2016 (Fig. 5).

A most likely cluster, and two secondary likely clusters were detected in a 14-year duration (Table 3). The most likely cluster included Jiujiang city (Wuning county), Nanchang city (An’yi and Wanli county), and Yichun city (Jing’an, Yifeng, Shanggao, Gao’an and Fengxin county). Wanli was in the plain states bordering on rivers and lakes, and the other counties attributed to Jiangxi northern hilly state. The expected case number was 85.05, while the observed case number was 896. The relative risk for the analysis was 11.89 (LLR = 1,346.95, *P* < 0.05). A total of 2,474,558 human beings were included with a time frame from 1/1/2013 to 31/12/2015. The first secondary cluster covered Shangrao city (Yushan, Shangrao, Xinzhou, Guangfeng, Hengfeng and Qianshan county) in the eastern part of Jiangxi northern hilly state, and the second secondary cluster covered Yichun (Fengcheng county), Ji’an (Yongfeng and Xingan county) and Fuzhou city (Linchuan, Jinxi, Zixi, Lichuan, Nancheng, Nanfeng, Guangchang, Yihuang, Chongren and Le’an county), of these counties attributing to the plain state bordering on rivers and lakes, Jiangxi northern hilly state and Wuyi mountainous hilly state, separately.

**Table 3:** Spatiotemporal clusters of hemorrhagic fever with renal syndrome cases in Jiangxi at the county level, 2005–2018.

	Most likely cluster	1st Secondary clusters	2nd Secondary clusters
Longitude (E)	28.71	28.49	27.39
Latitude (N)	115.18	118.04	116.24
Radius (km)	65.20	53.76	90.50
Time frame	2013/1/1 to 2015/12/31	2011/1/1 to 2013/12/31	2016/1/1 to 2018/12/31
Population	2,474,558	3,055,676	5,568,486
No. counties	8	6	13
Cluster counties	Wuning, Jingan, Anyi, Wanli, Fengxin, Yifeng, Shanggao, Gaoan	Yushan, Shangrao, Xinzhou, Guangfeng, Hengfeng, Qianshan	Fengcheng, Linchuan, Jinxi, Zixi, Lichuan, Nancheng, Nanfeng, Guangchang, Yihuang, Chongren, Le' an, Yongfeng, Xingan
Annual cases/100,000	12.10	4.20	1.80
Observed/expected	10.54	3.62	1.56
Relative risk	11.89	3.77	1.58
Log-likelihood ratio	1,346.95	220.18	26.09
*P*-value	*p* < 0.05	*p* < 0.05	*p* < 0.05

Most likely cluster: *p*-value < 0.05; Secondary cluster: *p*-value < 0.05.

## Discussion

In recent years, though an obvious decline in HFRS cases followed intensified control campaigns in China, HFRS would persist in China under the current control and prevention measures^23^. The results of this study also revealed that the amplitude and the magnitude of HFRS outbreaks in Jiangxi increased from 2005 to 2018. The susceptible population remained mainly in male farmers in the age groups of 16 to 60 years old, but the proportion of female HFRS cases was increasing.

Consistent with several studies^9,11,24^, the results of this study showed that the incidence of HFRS in Jiangxi had a major epidemic season (winter, November–January) and a minor seasonal peak (early summer, May–June) each year, which is corresponded to the epidemic peak of HTNV-type HFRS and SEOV-type HFRS, respectively^15^. Hereby, this study indicated that Jiangxi was a mixed epidemic area dominated by HTNV. The result differed from that of a previous virology study in 2002, which indicated that Jiangxi was a mixed epidemic area dominated by SEOV-type based on the amplification and sequencing results of hantavirus-positive mice^25^. A possible explanation was that environmental change, with the rapid urbanization process, could alter the composition of rodent species and increase the number of striped field mice in urban area^26^, causing that Jiangxi developed into a HTNV-type dominated mixed epidemic area. In addition, SEOV infections were more likely to be neglected because of the mild symptoms causing low rates of visiting doctors^27^. However, SEOV infection should not be ignored even though HTNV infection was dominated at present. Because unlike other hantaviruses, SEOV could easily spread throughout the entire colony when they were introduced^28,29^. Moreover, the host of SEOV, *R. norvegicus*, whose lifestyle differs greatly from that of *A. agrarius*, feeding on various food items from humans instead of field crops, are widely distributed in Jiangxi^30,31^, which is more likely to transmit the virus to humans compared with striped field mice. Unluckily, we are lack of the virus surveillance data until now. Therefore, more research efforts should be undertaken in animal hosts surveillance, causative agents detection, and latent infection investigation in the future in Jiangxi to implement targeted strategies of HFRS prevention and control.

The global model revealed that the distribution of HFRS cases in Jiangxi exhibited the characteristics of spatial aggregation. Subsequently, the LISA model and spatiotemporal scan analysis showed that the higher risk areas of the HFRS outbreak were mainly located in Jiangxi northern hilly state, spreading to Wuyi mountain hilly state as time advanced, but no HFRS cases were reported in Jiangxi southern mountainous state. This phenomenon might be affected by natural environmental factors, such as altitude. Some studies have revealed that HFRS occurrence was restricted to areas of altitude of below 200 m^15,32,33^. The average altitude ranges from 300 to 500 m in Jiangxi southern mountainous state, while it is flatter and lower in the middle and northern half. Moreover, though it was difficult to collect available data of the rodent host for our study, a past study in 1985 in Jiangxi indicated that *A. agrarius* was dominant rodent species in Jiangxi northern plain region, with no distribution in Jiangxi southern mountainous region^25^. This suggested that the distribution of HFRS cases was consistent with that of the rodent host. However, the higher incidence areas of HFRS were in Jiangxi northern hilly state instead of the low-lying plain state of bordering rivers and lakes, which was also different from the results of neighboring provinces^34,35^. This phenomenon might be explained by several factors. Firstly, the low-lying plain state was not only the Poyang Lake eco-economic zone, but also the political, cultural, and economic center of Jiangxi province with an urbanization process at an unprecedented pace over the past 15 years, which has reduced the risk of human-rodent contact because of the better environment^36–39^. Secondly, in 1998, the Yangtze river flooding occurred in the whole region, and then the project of “returning the farmland to the lake” was implemented in 2003, which caused the water level of Poyang lake to rise. The expansion of the wetland area might have induced that the rodent host shifted to Jiangxi northern hilly state with higher altitude^40,41^. Finally, to protect the ecological environment of Poyang lake, the implementation of “Mountain-River–Lake Program”, with the ecological concept of “mountain as source, river as connection flow, and lake as storage” 20 years ago has accelerated the local industrial transformation, and more and more local farmers worked in enterprises or engaged in technical production^42^. All the above factors in the region provided human beings with fewer opportunities for exposure to rodent hosts.

Some limitations should be considered when interpreting our findings. Firstly, the bias could exist in this study because the HFRS cases did not differentiate HTNV from SEOV infections, and came from the passive surveillance system called CISDCP. Secondly, social factors and urbanization level might have an important impact on the epidemic process of HFRS but were not included in the analysis due to data unavailable. Finally, specific species of rodent populations were not characterized in our study area.

In conclusion, this study has analyzed the spatiotemporal characteristics of HFRS comprehensively in Jiangxi from 2005 to 2018. We found that HFRS cases were aggregated in Jiangxi northern hilly state, spreading to Wuyi mountain hilly state as time advanced. Therefore, more interventions should be implemented for the prevention and control of HFRS in the future, including animal hosts surveillance, causative agents detection, medical training, and health promotion. Most importantly, further research efforts should be undertaken to explore the potentially influential factors and etiological surveillance in the next step to provide more scientific evidence for the prevention and control of HFRS.

## Data Availability

All data involved in the study are available from Q. Liu and S.E.C.
